# Evaluation of Marine Microalga *Diacronema vlkianum* Biomass Fatty Acid Assimilation in Wistar Rats

**DOI:** 10.3390/molecules22071097

**Published:** 2017-07-01

**Authors:** Cristina de Mello-Sampayo, Angela Paterna, Ambra Polizzi, Diana Duarte, Irineu Batista, Rui Pinto, Patrícia Gonçalves, Anabela Raymundo, Ana P. Batista, Luísa Gouveia, Beatriz Silva-Lima, Narcisa M. Bandarra

**Affiliations:** 1 Department of Pharmacological Sciences, iMed.ULisboa, Faculdade de Farmácia, Universidade de Lisboa, Lisbon 1649-003, Portugal; csampayo@gmail.com (C.d.M.-S.); apaterna@gmail.com (A.P.); ambra.polizzi@gmail.com (A.P.); rapinto@ff.ul.pt (R.P.); beatrizlima@netcabo.pt (B.S.-L.); 2 Division of Aquaculture and Upgrading, Portuguese Institute for the Sea and Atmosphere (IPMA, IP), Rua Alfredo Magalhães Ramalho, Lisbon 1495-006, Portugal; dduarte@gmail.com (D.D.); irineu@ipma.pt (I.B.); 3 Division of Modelling and Marine Resources, Portuguese Institute for the Sea and Atmosphere (IPMA, IP), Rua Alfredo Magalhães Ramalho, Lisbon 1495-006, Portugal; patricia@ipma.pt; 4 LEAF (Linking Landscape Environment Agriculture and Food), Research Center, Instituto Superior de Agronomia, Universidade de Lisboa, Tapada da Ajuda, 1349-017 Lisbon, Portugal; anabraymundo@isa.ulisboa.pt (A.R.); apbatista@gmail.com (A.P.B.); 5 LNEG_Bioenergy Unit, Estrada do Paço do Lumiar, 22, 1649-038 Lisbon, Portugal; luisa.gouveia@lneg.pt; 6 Interdisciplinary Centre of Marine and Environmental Research (CIIMAR/CIMAR), University of Porto, Rua dos Bragas 289, P 4050-123 Porto, Portugal

**Keywords:** *Diacronema vlkianum* supplementation, microalgae-biomass bioavailability, EPA, DHA, omega-3 index, cardiovascular benefits, tissues fatty acid profile

## Abstract

*Diacronema vlkianum* is a marine microalgae for which supposed health promoting effects have been claimed based on its phytochemical composition. The potential use of its biomass as health ingredient, including detox-shakes, and the lack of bioavailability studies were the main concerns. In order to evaluate the microalgae-biomass assimilation and its health-benefits, single-dose (CD1-mice) studies were followed by 66-days repeated-dose study in Wistar rats with the highest tested single-dose of microalgae equivalent to 101 mg/kg eicosapentaenoic acid + docosahexaenoic acid (EPA+DHA). Microalgae-supplementation modulated EPA and docosapentaenoic acid enrichment at arachidonic acid content expenditure in erythrocytes and liver, while increasing EPA content of heart and adipose tissues of rats. Those fatty acid (FA) changes confirmed the *D. vlkianum*-biomass FA assimilation. The principal component analyses discriminated brain from other tissues, which formed two other groups (erythrocytes, liver, and heart separated from kidney and adipose tissues), pointing to a distinct signature of FA deposition for the brain and for the other organs. The improved serum lipid profile, omega-3 index and erythrocyte plasticity support the cardiovascular benefits of *D. vlkianum*. These results bolster the potential of *D. vlkianum*-biomass to become a “heart-healthy” food supplement providing a safe and renewable source of bioavailable omega-3 FA.

## 1. Introduction

The importance and therapeutic value of omega 3 (n-3) polyunsaturated fatty acids (PUFA), especially eicosapentaenoic (EPA, 20:5n-3) and docosahexaenoic acid (DHA, 22:6n-3) is well recognized among the scientific community. Until now the major dietary source of these fatty acids (FA) has been seafood. Microalgae are new attractive sources of PUFA including long chain n-3 PUFA and due to the increased interest in and knowledge about microalgae production, they are now being considered as potentially relevant dietary elements with beneficial n-3/n-6 ratios [1].

The lipid content of the microalgae biomass can vary between 10% and 40% and under certain culture conditions it can reach 85% of its dry weight [2]. According to Behrens and Kyle [3], the largest fraction of total lipids (25–60%) produced by microalgae corresponds to the n-3 PUFA type of FA. It is important to stress that EPA and DHA are not produced by fish, but by marine microalgae, being accumulated along the food chain [4]. Thus, the direct consumption of microalgae by humans is a viable alternative source of n-3 PUFA [5,6]. This is a feasible change, not only for fans of a vegetarian diet, but also for those who are not fond of fish, as it allows to include n-3 PUFA in diet without the typical fish consumption drawbacks (odour/taste) [7]. That is why, in recent years, commercial production of n-3 PUFA-rich oils from microalgae has increased in such a way that in the U.S. the utilization of dietary supplements with microalgae oils is reaching 84% in infant formulas [8].

*Diacronema vlkianum* (family Pavlovaceae), a widespread marine green microalgae, is still scarcely studied although the phytochemical composition has been thoroughly analysed [9,10,11,12]. The chemical composition of *D. vlkianum* is very sensitive to the life cycle phase and growth temperature, with considerable increases in biomolecule production being observed at 18 °C during late exponential phase [10]. In stationary phase the contents of sterols, fatty acids and total carotenoids were the highest [11].

*D. vlkianum* richness in n-3 PUFA, mainly eicosapentaenoic acid (EPA, 20:5n-3) and also docosahexaenoic acid (DHA, 22:6n-3), that are accumulated as oil droplets in prominent lipid bodies in the cell, makes this microalgae a potentially promising source for the aquaculture and food industries as a valuable alternative to fish oil sources of n-3 PUFA, while also supplying several sterols, among which β-sitosterol always figures prominently, in addition to tocopherols, carotenoids, other colouring pigments and other nutraceuticals [9,10,11,12]. When the *D. vlkianum* fatty acid profile, pigments profile, and mineral content is compared to *Spirulina (Arthrospira) maxima* [9], both algae are rich sources of protein, but the advantage of *D. vlkianum* regarding the n3/n6 ratio (4.1 vs. 0.1), the n3-PUFA content (100 times higher), pigment content (2.4% vs. 0.9%) and other minerals, while providing lower amounts of sodium and potassium is notorious.

Although microalgae have been consumed since ancient times, only few of them, like *Spirulina*, *Chorella*, and astaxanthin-rich extracts derived from *H. pluvialis*, have undergone a series of toxicological tests to prove their safety as food items [9]. The successful authorization of microalgae as food or food ingredients broadens the perspectives for a wider inclusion of these valuable microorganisms in the human diet, food- or even the pharmaceutical-industry. In the case of *D. vlkianum*, the health promoting effects have been claimed based solely on its phytochemical composition, yet its claimed health benefits have not been demonstrated. *D. vlkianum*’ biomass potential inclusion in detox-shakes based on its health benefits (fatty acid profile, pigments profile, mineral content) has prompted us to study the bioavailability and consequent health benefits of regularly ingested *D. vlkianum*-biomass as a whole. The assimilation of *D. vlkianum*-biomass intake, at the highest dose tested (selected in a preliminary single dose, dose finding study in mice), was studied in rats by tracking the fatty acid content of several organ-tissues, including the brain. Additionally, the health benefits of regularly ingested *D. vlkianum*-biomass were assessed on plasma biomarkers and fatty acid composition of relevant target tissues such as liver, erythrocytes, heart, kidney, adipose tissues (subcutaneous and visceral fat) and brain.

## 2. Results

### 2.1. Diet Fatty Acid Profile

The obtained FA profile of *D. vlkianum*-biomass and chow was used to estimate the FA profile of each diet (Table 1). Generally, both diets, supplemented or not, had the same relative proportion of saturated fatty acids (SFA), monounsaturated fatty acids (MUFA) and polyunsaturated fatty acids (PUFA). PUFA were the major class of FA in both diets, but the diet supplemented with the microalgae was richer in n-3 FA content (16%) compared to the 3% content of the regular diet. *D. vlkianum* was rich in those n-3 FA like EPA, DPA (docosapentaenoic acid, 22:5n-3) and DHA, and its inclusion in the diet resulted in an increased intake of 10 times EPA, 100% DPA and four times the DHA compared with regular diet. Of relevance to note is that regular diet lacks DPA and linoleic acid (18:2n-6) is its major PUFA.

### 2.2. Animal Dosing Studies

In both studies, mice single dose and rat repeated dose, all animals survived for the duration with all dose treatments (single and repeated oral administration). The tested single doses of *D. vlkianum*-biomass—0.25, 1.25 and 2.50 g/kg—corresponded to a daily intake of 0.33%, 1.65% and 3.24% dried microalgae (relative to global daily intake: chow (g) + microalgae (g)), being the animals exposed to marine bioproducts in non-purified form (microalgae-biomass) including an extra amount of FA (equivalent to an EPA+DHA dose of 10.1, 50.6 and 101 mg/kg). During the five days that followed the single-dose administration in mice no signs of toxicity (including body-weight, food and water intake variations) and no changes in animal behaviour were observed when compared to the control group. The macroscopic examinations showed no differences between treated and control animals, as well, and no other signs of toxicity were observed.

The highest tested dose in mice was chosen to proceed with *D. vlkianum*-biomass bioavailability studies in rats; during 66 days of daily supplementation with 2.5 g/kg of microalgae no differences (*p* < 0.05) among animals feed with *D. vlkianum*-biomass diet and those feed with regular diet concerning feed, water intake and body-weight were detected. Feeding 3.24% *D. vlkianum*-biomass (chow (g) + microalgae (g)) or a regular diet (chow) for 66 days resulted in an average body-weight gain of 88 g in both animal groups (body-weight 383.7 ± 26.8 g and 384.8 ± 56.9 g, respectively), though the *D. vlkianum*-biomass diet provided an extra daily fat intake of 0.35 g/kg (chow + microalgae) but with improved n-3/n-6 ratio, from 0.03 to 0.19 (Table 1). The maximum tested dose in mice corresponded to a human equivalent dose of 0.65 g/day EPA+DHA while in rats it corresponded to a dose of 1.27 g/day EPA+DHA [13].

### 2.3. Biochemical Parameters

The glycaemia and plasma biomarkers recorded during the repeated dose-study (D16, D30, D66) for both diet groups are shown in Table 2. At the beginning of the repeated-dose study (D0) the fasting blood glucose levels of both rat groups was not statically different (regular diet: 65.8 ± 2.1 mg/dL; *D. vlkianum*-supplemented: 61.6 ± 2.6 mg/dL). The same pattern of glucose levels variation was observed during the 66 days study period, as no significant changes on fasting glycaemia were observed between and within each diet group (Table 2).

The diet induced changes in the lipids profile were only detectable with significant differences following 66 days to diet exposure (Table 2) and show different trends regarding cholesterol and low-density lipoprotein cholesterol (LDL-C) content, namely: A maintained cholesterol level is observed in *D. vlkianum*-supplemented, which is significant lower and contrasts to the increased trend of total cholesterol content observed in the regular diet group along the study; animals supplemented with microalgae-biomass had a significant decrease in LDL-C along the study. The same variation pattern of high-density lipoprotein cholesterol (HDL-C), very low-density lipoprotein cholesterol (VLDL-C), triglycerides (TAG) and total lipids were observed between both studied dietary groups. No significant changes on analysed renal functional parameters (creatinine and urea) and hepatic biomarkers (alkaline phosphatase (ALP), alanine aminotransferase (ALT) and aspartate aminotransferase (AST)) have been observed along the study and across dietary treatments.

The volume increase (plasticity) properties of erythrocytes, assessed through osmotic stress on day 16, day 30 and day 66 of diet supplementation, are shown in Table 2. Significant changes were only detected at day 66, being erythrocytes of *D. vlkianum*-supplemented animals less prone to lysis (*p* < 0.005) at hypotonic conditions than those of animals under regular diet.

### 2.4. Cells and Tissue Fatty Acid Composition

The analysis of cells and tissues FA composition (Table 3) revealed no significant differences in the brains of control and microalgae group animals. In erythrocytes and liver of *D. vlkianum*-supplemented rats a significant decrease of arachidonic acid (AA) levels occurred compared to regular diet-fed animals, with a similar tendency, although slight, being observed in the kidney, visceral fat and brain. EPA content was significantly higher in most of the analysed cells and tissues of *D. vlkianum*-supplemented compared with regular diet fed animals (EPA content increased 2.2, 2.3, 2.6, 3.5, 5.5-fold in erythrocytes, liver, heart, subcutaneous and visceral fat) with exception of kidney and brain. DPA content increased in most of the tested tissues being significantly higher in liver and erythrocytes. No significant changes on DHA content occurred after *D. vlkianum*-supplementation in all analysed cells/tissues compared to control. The n-3 PUFA content increased in liver and heart (*p* < 0.05) and a similar tendency was observed in subcutaneous fat, kidney and erythrocytes of the *D. vlkianum*-supplemented animals. Those observed changes led to a significant increase of n-3/n-6 ratio in erythrocytes, liver and subcutaneous fat of *D. vlkianum*-supplemented rats.

### 2.5. PCA on Analysed Cell and Tissues Fatty Acids Profile

**Figure 1:**
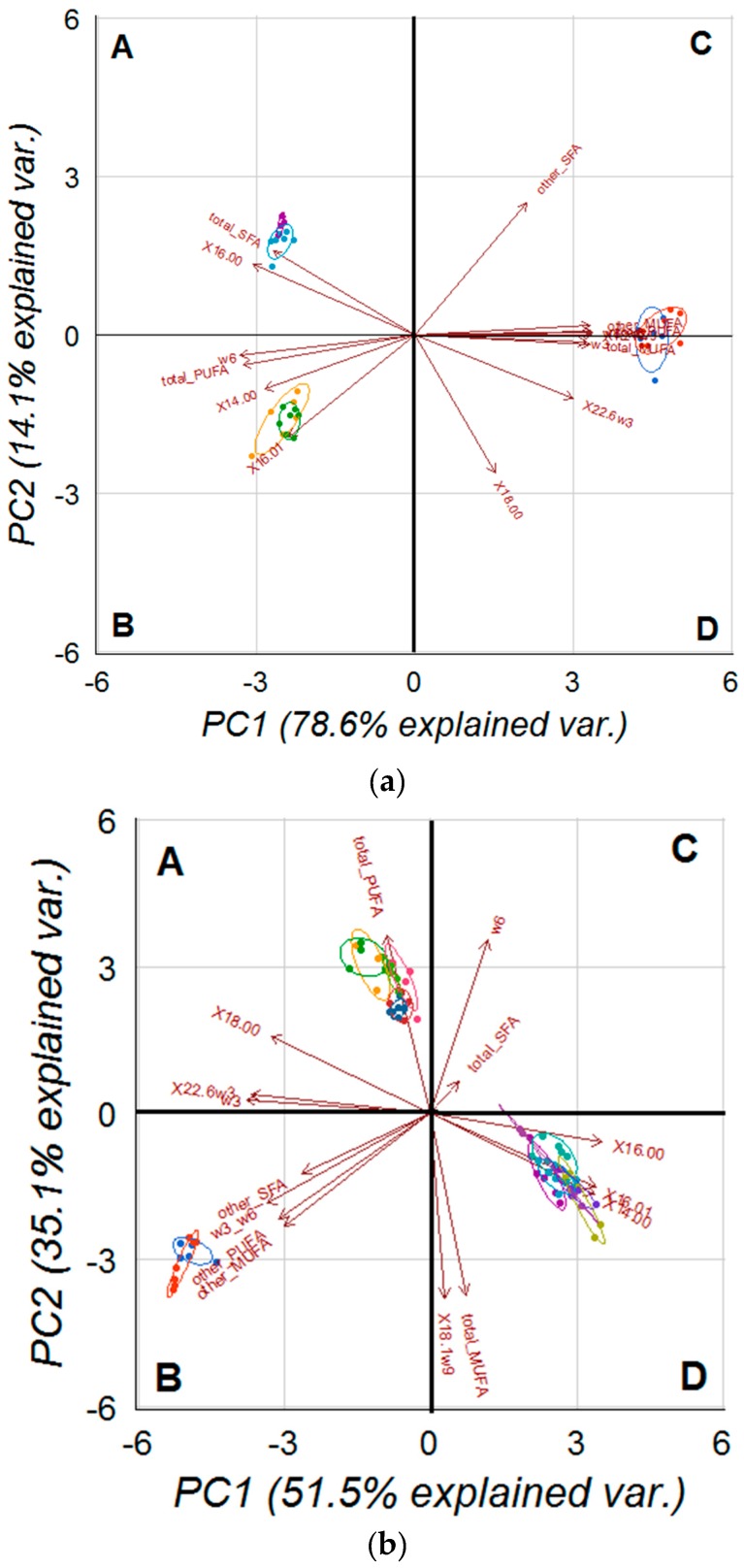
Loading plot of the first and second principal components of the pooled FAME and component’s score vectors of liver, erythrocytes, heart, kidney, subcutaneous and visceral fat and brain from Wistar rats fed normal (CG) and *D. vlkianum*-biomass supplemented (MG) diets. Legends: (**a**) brain (CG) (●), brain (MG) (●), liver (CG) (●), liver (MG) (●), RBC (CG) (●) and RBC (MG) (●); (**b**) brain (CG) (●), brain (MG) (●), heart (CG) (●), heart (MG) (●), kidney (CG) (●), kidney (MG) (●), liver (CG) (●), liver (MG) (●), RBC (CG) (●), RBC (MG) (●), subcutaneous fat (CG) (●), subcutaneous fat (MG) (●), visceral fat (CG) (●), visceral fat (MG) (●).

Figure 1 displays the projection of PC1 and PC2 in the plane using the determined percentages of fatty acids. In Figure 1a it is shown the principal component analysis (PCA) of three conventional tissues (liver, erythrocytes and brain) while in Figure 1b it is shown the PCA for all seven tissues studied in the present study (liver, erythrocytes, heart, kidney, subcutaneous and visceral fat and brain). For PCA of the three tissues (a), both principal components (PCs) combined accounted for 92.7% of the total variance whereas in the PCA of the seven tissues (b) it accounted for 86.6% of the total variance. Variables with positive/negative loading either on PC1 or PC2 change depending on PCA. On PCA of all the seven studied tissues, PC1 was characterized by variables with positive loadings, such as 14:0 (0.316), 16:0 (0.329), 16:1 (0.317), 18:1n-9 (0.027), and by variables with negative loadings, such as 18:0 (−0.306) and 22:6n-3 (−0.345). PC2 was positively defined by 18:0 (0.180), 22:6n-3 (0.043), and negatively defined by 14:0 (−0.189), 16:0 (−0.068), 16:1 (−0.170) and 18:1n-9 (−0.429).

For the PCA of liver, erythrocytes and brain the projection of the scores in the PC1 versus PC2 in plane (Figure 1a) distinguished three well-defined clusters matching the three analysed tissues: erythrocytes (located in quadrant A), liver (located in quadrant B) and brain (located between quadrant C and D). Remarkably, the erythrocytes cluster showed slightly less dispersion than the brain and the liver clusters. For the PCA of all the seven tissues analysed the projection of the scores in the PC1 versus PC2 in plane (Figure 1b) also grouped the tissues in three well-defined areas, but setting apart brain (located in quadrant B) from the other two groups of tissue clusters: heart, liver, erythrocytes (located in quadrant A), kidney, subcutaneous and visceral fat (located in quadrant D). Again, the erythrocytes cluster showed the least dispersion. Discrimination of both dietary groups within each tissue was unachievable during the study period, but it can be clearly delimited an additional small cluster for liver, for erythrocytes and for brain, especially in the second, corresponding to the two months diet supplementation with *D. vlkianum*.

## 3. Discussion

*D. vlkianum* microalgae best production practices and phytochemical characterization have been widely studied [9,10,11,12]. Based on its phytochemical composition (fatty acid profile, pigments profile, mineral content), claims of health promoting effects have been made, but have not been demonstrated, yet. *D. vlkianum* biomass potential inclusion in detox-shakes and use as food ingredient based on its supposed health benefits, plus the industry doubts about the nutritional value of algae biomass used as a whole, has prompted us to study the bioavailability and consequent health benefits of regularly ingested microalgae-biomass. The assimilation of ingested *D. vlkianum*-biomass, at the highest dose tested in mice, was studied in rats by tracking the fatty acid content from the liver (the key metabolic organ) to erythrocytes (representative of systemic bioavailability), the heart, kidney, adipose tissues (subcutaneous and visceral fat) and the brain (the most conservative organ in terms of FA uptake). Additionally, the health benefits of ingested *D. vlkianum*-biomass were regularly assessed on plasma biomarkers and the fatty acid composition of relevant target tissues analysed.

Dried *D. vlkianum*, with FA profile in the published range [9,11,12], supplied the treated animals with marine biomolecules in non-purified form (microalgae-biomass) including an extra daily FA intake of 0.35 g/kg (chow + microalgae) with improved diet n-3/n-6 ratio (from 0.03 to 0.19) at the highest tested dose (3.24% dried microalgae relative to daily intake). The maximum *D. vlkianum*-biomass tested dose, equivalent to an EPA + DHA dose of 101 mg/kg, corresponded to a human equivalent dose of 0.65 g/day EPA + DHA if based on mice dosing, while in rats it corresponded to a dose of 1.27 g/day [13]. EPA+DHA human daily dose up to 1 g/day is recommended in secondary cardiovascular disease (CVD) prevention [14,15,16]. It is worth noting that all *D. vlkianum* tested doses and dose-regimes induced no toxicity or intolerance effects and no changes in hepatic and renal safety biomarkers were noted during the 2-months study in rats. A benign safety profile for *D. vlkianum*-biomass, in doses close or exceeding the human recommended EPA + DHA daily doses, is therefore suggested by these results.

Additionally, the animals showed no variation in weight gain and in food intake among diet treatments although *D. vlkianum*-treated animals had additional caloric intake from the microalgae supplement. It has been suggested that EPA rich diets might have strong effects on body weight reduction due to increased energy expenditure [17], which, taking into account the *D. vlkianum* richness in EPA, could explain the absence of body weight gain in regard to the caloric intake. One may argue that non-absorption of ingested microalgae-biomass is a reasonable explanation of the unchanged body weight. However, the observed change in PUFA contents of some tissues in rats fed *D. vlkianum*-supplemented diet for two months (for example: Increased level of 14:0, EPA and DPA—three FA components of *D. vlkianum*) particularly in those absent in regular diet, as it is DPA, support the digestibility of this microalgae, including the absorption of their molecules/FA. Similar to these results, the rats skin fatty acids pattern has also been modulated into richest n-3—EPA, DPA, DHA—through diet supplementation with a rich source of n-3 PUFA, for 3 months, [18]. Furthermore, the present study revealed a high susceptibility of the liver and erythrocytes FA content to *D. vlkianum*-supplementation while the heart the kidney and adipose tissue were less influenced and the brain unaffected. These findings are in line, and add up, to results described in hamsters fed with DHA- and EPA-rich diets [19], suggesting a selectively mobilization of FA toward organs with specific requirements, such as liver and heart, instead of being directed to energy storage tissues (like adipose tissue), and a clear separation of the brain from the remaining tissues, as revealed by PCA analysis.

The reduction of lipid blood parameters, like the level of total cholesterol, LDL-C in rats supplemented with *D. vlkianum*-biomass is in accordance with previous reported results in mice and hamsters fed with PUFA rich diets [19,20]. Unlike other studies reporting changes on HDL-C levels by several types of PUFA diets [17,21], microalgae biomass did not affect HDL-C levels. There is evidence that n-3 PUFA can affect lipid metabolism not only in the liver but also in the intestine, through induced expression of the intestinal cholesterol transporter and bile acid transporter that promote cholesterol excretion in faeces [22], which could have contributed for the observed systemic cholesterol decrease. Furthermore, the observed decrease in systemic total cholesterol and LDL-C, besides the change in FA profile of analysed tissues, reinforces the microalgae-biomass molecules assimilation.

In erythrocytes the decreased AA content and the two-fold enrichment in EPA and DPA by *D. vlkianum*-supplementation are in line to other studies where a rapid accumulation of EPA in human erythrocytes after these n-3 PUFA supplementation is shown [23]. These FA changes resulted in an increase in the n-3/n-6 ratio coincident with the observed beneficial effect on erythrocytes plasticity of rats fed *D. vlkianum*-diet for two month (D66), are in line with rat’s erythrocytes lifetime [24,25], and with the proposed use of blood cells as a long term FA balance marker with no influence of recent dietary fat intake [26]. Consequently, the omega-3 Index (content of EPA + DHA expressed as a percentage of total FA in erythrocytes) of animals fed with *D. vlkianum* biomass increased to 5.1%. This index is a biomarker of risk for death from CVD [27]: values between 4% and 8% indicate an intermediate cardio-protection and above 8% greatest cardio-protection. Total cholesterol and LDL-C, well-known lipid biomarkers of risk for death from CVD, were lower in animals fed *D. vlkianum*-diet after 2 months of supplementation, again suggesting a potential cardiovascular protective role by *D. vlkianum*-biomass supplementation. The observed heart enrichment in EPA following *D. vlkianum*-supplementation, which also occurred in rats supplemented with n-3 PUFA [28], reinforces the cardiovascular beneficial effects that may derive from the use of *D. vlkianum*-biomass as supplement. Additionally, the EPA, DPA and DHA incorporation at expense of the AA could positively impact inflammation, as lees substrate is available for the synthesis of pro-inflammatory biomarkers, which has also been observed in mice fed alga [29].

The liver FA content was strongly modified in rats fed *D. vlkianum*-biomass and similar to those observed in erythrocytes, added by a significant increase in n-3 PUFA. Similar results were reported for rats supplemented with oil from *Nannochloropsis* or *Arthrospira* and for pigs supplemented with microalgae [30,31,32]. These results suggest that the microalgae content in FA importantly modulate the FA composition of tissues like the blood and liver, and agree with Bandarra et al.’s [19] observation of great liver susceptibility to dietary n-3 FA compositions. Regulation of lipid and lipoprotein metabolism is a major liver function, but this organ is not committed to store lipids [22], which can be hepatotoxic. In this study, despite the extra fat intake by rats fed *D. vlkianum*-biomass, no changes occurred in the hepatotoxicity biomarkers, hence excluding the induction of any possible liver injury.

The subcutaneous and visceral fat were enriched in EPA following *D. vlkianum*-diet. Although adipose tissue core function is to accumulate fat, PUFA enrichment of adipocytes normally is very low (0.1%, EPA or DHA) [33], in line with the lack of phospholipids in fat storage tissues [30] explains the observed PUFA low levels. The kidney of rats fed microalgae-biomass showed a 14:0 enrichment but unaltered PUFA content pointing to a non-preferential tissue for n-3 PUFA incorporation. Brain was the only tissue with FA composition unaffected by *D. vlkianum*-supplementation. Identical brain results have been obtained with higher doses of EPA/DHA in rats and pigs [34,35]. Typical of this organ [36], the brain presented the highest content in DHA among all analysed tissues, and sets a clear separation of brain from the analysed tissues by PCA of FA pooled data, similar as previously reported [19]. The brain phospholipid richness (high levels of phosphatidylethanolamine, phosphatidylserine and phosphatidylcholine), explains the high content of brain in DHA [30].

In our study, DHA content remained unchanged in all tissues, which suggest that the DHA level present in maintenance chow seems to be enough to balance the metabolic process where DHA is involved: partial β-oxidation and the retroconversion to EPA [28].

Although the public general idea, as well as the industry one, may consider the microalgae biomass just a source of non-absorbable fibre, this study has shown that at least microalga FA, among possible other bioactive components, are absorbable, modulating FA content of target organs with health beneficial effects at daily doses corresponding to human equivalent dose [13] of 24,2 g microalgae, which seems a sustainable and reasonable amount of *D. vlkianum* for a 60 kg person to consume on a daily basis at once or even divided in three doses of 8 g per day. Furthermore, if incorporated in the diet, *D. vlkianum* microalgae could be seen as one additional source of omega-3 fatty acids, to be used in isolation or together with other sources like fish.

## 4. Materials and Methods

### 4.1. Microalgae Biomass Production

*Diacronema vlkianum*, obtained from the Mary Parke Collection (Plymouth Laboratory, Plymouth, UK), was cultivated through inoculation of axenic microalgal cultures in appropriated growth medium, Wallerstein/Míquel medium (3:1, in filtered seawater with 35% salt), at 18 °C and low light conditions (150 μE m^−2^ s^−1^) in 1 L airlift bioreactors before scaled-up until 100 L polyethylene bags bioreactors (40 cm Ø), with bubbling filtered air (without CO_2_ addition). At middle stationary growth phase, microalgae-biomass harvesting was performed without flocculation by simply stopping agitation and concentration by centrifugation and freeze-drying.

### 4.2. Animals

Male CD-1 mice and Wistar rats, from Harlan Laboratories (Barcelona, Spain), were fed with maintenance diet A04 (SAFE, Augy, France) and water ad libitum. All experiments were carried out in accordance with relevant legislation in force at time regarding the protection of animals used for experimental and other scientific purposes, the European Community Council Directive of 24 November 1986 (86/609/EEC) and the National Law of 6 July 1992 (DL. Nº 129/92) and of 23 October 1992 (Portaria Nº 1005/92) for minimizing animal suffering and to use only the number of animals necessary to produce reliable results.

### 4.3. Animal Studies

#### 4.3.1. Single Dose Mice Study

Preliminary dose-finding studies were conducted in male mice weighting 27 ± 3 g and followed by a repeated-dose study in Wistar rats for *D. vlkianum*-biomass bioavailability assessment purposes. Mice randomly divided in groups of 5 mice/dose, were gavage with single-doses of 0, 0.25, 1.25 and 2.50 g/kg of microalgae biomass suspended in water and fed with maintenance diet and water ad libitum. Behaviour and clinical signs (morbidity and mortality) were monitored for 2 h after administration and twice a day during the following 5 days. Body weight, feed and water intake were recorded one-week prior (baseline) and monitored daily during the study. Mean body-weight and body-weight changes were calculated. Final body weights (fasted) recorded prior to necropsy.

#### 4.3.2. Repeated Dose Rat Study 

For microalgae-biomass bioavailability studies a repeated-dose protocol, using the highest tested dose in mice, was undertaken in Wistar rats weighing 292.8 ± 22.3 g, randomly divided into two groups fed with maintenance diet and water ad libitum, were supplemented by gavage, for 66 days (9 weeks), with microalga (2.5 g/kg; *n* = 15) or with an equivalent volume of water (control, *n* = 14). Body-weight, feed and water intake were recorded one-week pre-treatment (baseline) and subsequently on a daily basis. The final body weights (fasted) were recorded prior to the scheduled necropsies (D16 (four animals/group), D30 (four animals/group) and D66 (six/seven animals per control/test group)). Overnight fasting blood glucose levels were periodically monitored (D0, D14, D28 and D60) by measuring glycaemia on tail blood samples (Accutrend Plus system, Roche, Lisbon, Portugal).

At the end of either the single-dose study or the schedule repeated-administration period, a complete necropsy was conducted on all animals which included examination of the external surface, the cranial, thoracic, abdominal and pelvic compartments, including viscera as previously described [24]. In addition, rats’ liver, kidney, heart, brain, visceral and subcutaneous-fat samples were frozen for subsequent FA analysis (repeated-dose study).

### 4.4. Analysis of Erythrocytes and Plasma Biochemical Parameters

At scheduled post-dose day, fasting blood samples were collected in lithium-heparin tubes, the plasma obtained by centrifugation (1500 g/10 min) and the separated erythrocytes washed twice with 0.9% NaCl. All aliquots were flash-frozen in liquid nitrogen, stored at −80 °C for further analyses. Biochemical analysis of plasma total cholesterol, high-density lipoprotein cholesterol (HDL-C), low-density lipoprotein cholesterol (LDL-C), triglycerides (TAG), creatinine, urea, total proteins, alkaline phosphatase (ALP), aspartate aminotransferase (AST) and alanine aminotransferase (ALT) were done using standard diagnostic test kits from Roche Diagnostics (Mannheim, Germany) in a Modular Hitachi Analytical System (Roche Diagnostics). Very low-density lipoprotein cholesterol (VLDL-C) and total lipids were computed according to Friedewald et al. [37] and Covaci et al. [38] formulas, respectively. Blood samples were also collected in EDTA tubes for the evaluation of erythrocytes fragility by the osmotic fragility test using the serial dilution method. Erythrocytes fragility was expressed as the hemolysis rate obtained in hypotonic saline buffer (0.4% NaCl).

### 4.5. Analysis of Fatty Acid Composition

Fatty acids methyl esters (FAME) were prepared according to Bandarra et al. [39]. Samples were lyophilized (−60 °C and 2.0 hPa) to a constant weight. The FA methyl esters (FAME) preparation was carried out under acid conditions according to Donato et al. [11] using 0.3 g of freeze dried *D. vlkianum*-biomass, chow, and tissues except for fat samples where 0.1 g was used instead. FAME were concentrated to a final volume of 25 μL in n-heptane, and 2 μL of the sample was injected on a capillary DBWax capillary column (30 m × 0.25 mm ID × 0.25 μm film thickness; J&W Scientific, Agilent, Santa Clara, CA, USA) in a CP-3800 gas chromatograph (Varian, Palo Alto, CA, USA) equipped with a flame ionization detector. The injector and detector temperatures were set at 250 °C. Adequate separation was obtained over a 40-min period, with 5 min at 180 °C, followed by an increase of 4 °C/min until 220 °C, and kept at this temperature for 25 min. Authentic standards were used for fatty acid identification. Individual fatty acids were expressed as percentage of the total fatty acids.

### 4.6. Statistical Analysis

Data, expressed as mean ± standard deviation, was analysed by Student *t*-test followed by Holm-Bonferroni post-tests at *p* < 0.05. The principal component analysis (PCA) was applied in which the maximum variability of the data was explained in a reduced variable set [40]. The PCA aimed to correlate the relative differences among tissues in relation to their overall fatty acid composition. For that purpose, the PCA was carried out using a proportion of different fatty acids from liver, erythrocytes, heart, kidney, subcutaneous and visceral fat, and brain. Only fatty acids common to all tissues were included in the multivariate analysis. The PRINCOMP procedure of R was applied after data standardization and PCAs were based on the correlation matrix. PCA data analyses, including the figures and the models presented, were performed on R 3.3.1 [41] and using packages “ggplot2” and “ggbiplot” [42].

## 5. Conclusions

*D. vlkianum* oral supplementation modulated the PUFA content of target tissues and confirmed the assimilation of microalgae-biomass, particularly DPA augmentation in erythrocytes and liver, EPA enrichment of erythrocytes, liver, heart and adipose tissues. Liver, erythrocytes and heart were separated from kidney and adipose tissues, being brain set apart from all other tissues by PCA suggesting the existence of an individual signature for FA deposition. Furthermore, an improved serum lipid profile, omega-3 index and erythrocytes plasticity indicate a *D. vlkianum* cardio-protection due to PUFA-enrichment, mainly EPA. Yet, the fact these healthy effects result from the intake of the whole biomass, and therefore possibly could be attributed to many bioactive molecules, needs further research. These results bolster the *D. vlkianum* potential to become a “heart-healthy” food supplement providing a safe, sustainable and renewable source of bioavailable omega-3 FA.

## Figures and Tables

**Table 1:** Fatty acid profile (mg/kg of animal body-weight) of rats’ daily diets: regular diet (fed chow) and *Diacronema vlkianum* dose (2.5g/kg) with regular diet. Percentage (%) represents each FA ratio of total ingested FA on each diet treatment.

Fatty Acids	Regular Diet		Regular Diet + Microalga	
	mg/kg	(%)	mg/kg	(%)
14:0	8.9 ± 0.4	0.5	61.0± 1.0	3.2
16:0	323.7 ± 16.2	19.3	359.0± 11.2	17.6
18:0	32.1 ± 1.6	1.9	32.4± 1.1	1.6
Other SFA	15.7 ± 0.8	0.9	17.6 ± 0.7	0.9
Σ SFA	380.3 ± 19.0	22.7	470.0 ± 14.0	23.2
16:1	7.5 ± 0.4	0.4	68.1 ± 1.0	3.6
18:1	305.8 ± 15.3	18.2	312.1 ± 10.3	15.2
Other MUFA	37.3 ± 1.9	2.2	42.1 ± 1.6	2.1
Σ MUFA	350.5 ± 17.5	20.9	422.3 ± 12.8	20.8
18:2n-6 (LA)	835.3 ± 41.8	49.8	836.5 ± 28.9	40.6
20:4n-6 (AA)	2.2 ± 0.1	0.1	7.0 ± 0.1	0.4
Other n-6	82.0 ± 2.8	4.9	109.6 ± 1.1	5.5
18:4n-3	0.0 ± 0.0	0.0	28.0 ± 0.2	1.5
20:5n-3 (EPA)	7.5 ± 0.4	0.4	87.8 ± 0.8	4.7
22:5n-3 (DPA)	0.0 ± 0.0	0.0	16.3 ± 0.5	0.9
22:6n-3 (DHA)	7.5 ± 0.4	0.4	28.4 ± 0.4	1.5
Other n-3	14.9 ± 2.0	0.9	20.9 ± 2.5	1.0
Σ PUFA	947.1 ± 47.4	56.6	1134.4 ± 33.2	55.9
n-3/n-6	0.03		0.19	

SFA: saturated FA; MUFA: monounsaturated FA; PUFA: polyunsaturated FA; Average results ± standard deviation.

**Table 2:** Effect of *Diacronema vlkianum* biomass supplementation on rat’s plasma biomarkers, glycaemia and erythrocytes plasticity.

Biomarker	Regular Diet			*D. vlkianum*-Supplemented		
	D16	D30	D66	D16	D30	D66
Glycaemia ^1^ (mg/dL)	72.4 ± 2.3	64.6 ± 1.1	68.7 ± 2.2	66.5 ± 2.7	60.2 ± 1.9	61.4 ± 2.4
Total Cholesterol (mg/dL)	47.0 ± 3.1	48.0 ± 3.9	58.2 ± 4.2	48.0 ± 4.0	48.0 ± 4.4	48.6 ± 1.3 **^#^**
HDL-C (mg/dL)	6.8 ± 0.9	9.3 ± 2.8	14.0 ± 1.1 **	6.3 ± 1.0	9.8 ± 1.3	12.1 ± 0.5 **
LDL-C (mg/dL)	26.6 ± 5.5	23.6 ± 2.9	28.0 ± 3.3	28.6 ± 3.3	23.8 ± 4.7	19.3 ± 1.7 *^,**#**^
VLDL-C (mg/dL)	13.7 ± 2.9	15.2 ± 1.1	16.2 ± 1.2	13.2 ± 2.3	14.4 ± 1.0	17.2 ± 1.3
TAG (mg/dL)	68.5 ± 14.6	76.0 ± 5.4	81.0 ± 6.0	65.8 ± 11.5	72.5 ± 4.8	85.9 ± 6.7
Total Lipids (mg/dL)	291.8 ± 16.9	302.8 ± 10.8	320.9 ± 12.8	289.2 ± 16.6	298.2 ± 8.2	316.6 ± 9.4
Erythrocytes (% Hemolysis 67mM)	31.0 ± 2.4	27.0 ± 5.5	29.4 ± 2.5	32.8 ± 1.8	23.3 ± 3.9	22.1 ± 0.3 **^##^**
Creatinine (mg/dL)	0.59 ± 0.02	0.49 ± 0.01	0.48 ± 0.02	0.50 ± 0.02	0.47 ± 0.03	0.47 ± 0.01
Urea (mg/dL)	34.5 ± 4.7	31.3 ± 0.5	31.2 ± 1.2	28.8 ± 1.7	33.8 ± 1.9	35.6 ± 2.4
AST (U/L)	119.0 ± 11.2	113.5 ± 10.5	113.5 ± 14.4	130.0 ± 27.1	99.5 ± 9.2	108.1 ± 8.6
ALT (U/L)	8.0 ± 0.9	8.3 ± 1.2	7.2 ± 1.1	5.3 ± 1.0	5.0 ± 0.4	6.3 ± 1.0
ALP (U/L)	59.3 ± 5.3	71.3 ± 9.5	57.2 ± 4.3	68.3 ± 10.6	81.3 ± 3.7	68.6 ± 6.8

*n* = 4 per group on D16 and D30; *n* = 6/7 on D66 for control/*D. vlkianum* group; values are mean ± SEM * *p* < 0.05 D16vsD66; ** *p* < 0.005 D16vsD66; ^#^ *p* < 0.05 *D. vlkianum*-supplemented vs. control; ^##^ *p* < 0.01 *D. vlkianum*-supplemented vs control; ^###^ *p* < 0.005 *D. vlkianum*-supplemented vs. control; ^1^ Glycaemia was measured on D14, D28 and D60; VLDL-C=1/5 |TAG|; Total Lipids = |total chol| × 1.12 + |TAG| × 1.33 + 148.

**Table 3:** Fatty acid profile (%) of erythrocytes and tissues (liver, heart, kidney, subcutaneous and visceral fat and brain) at D66 of rats’ supplementation with *Diacronema vlkianum* (*n* = 6/7 per control (CG)/microalgae (MG) group).

	Erythrocytes		Liver		Heart		Kidney		Subcutaneous Fat		Visceral Fat		Brain	
Fatty acids	CG	MG	CG	MG	CG	MG	CG	MG	CG	MG	CG	MG	CG	MG
14:0	0.26 ± 0.04	0.30 ± 0.02	0.35 ± 0.12	0.34 ± 0.04	0.34 ± 0.17	0.39 ± 0.11	1.89 ± 0.20	2.18 ± 0.26 *	1.99 ± 0.32	2.21 ± 0.25	2.23 ± 0.10	2.38 ± 0.12	0.12 ± 0.03	0.12 ± 0.03
16:0	24.3 ± 0.72	24.3 ± 0.63	19.7 ± 0.96	19.8 ± 0.76	14.8 ± 2.73	14.8 ± 4.02	27.7 ± 1.36	28.9 ± 1.51	27.0 ± 1.80	27.2 ± 1.25	29.9 ± 0.95	30.8 ± 2.26	13.6 ± 0.96	13.1 ± 1.01
18:0	12.9 ± 0.94	13.0 ± 0.62	16.3 ± 1.13	17.1 ± 1.40	15.6 ± 2.51	15.7 ± 0.69	7.76 ± 1.02	7.23 ± 0.56	2.84 ± 0.48	2.84 ± 0.23	2.31 ± 0.65	2.75 ± 0.39	16.7 ± 0.73	16.8 ± 0.88
Other SFA	3.20 ± 0.56	3.14 ± 0.37	0.30 ± 0.02	0.29 ± 0.03	1.41 ± 1.05	0.69 ± 0.44	1.08 ± 0.16	1.08 ± 0.11	0.49 ± 0.17	0.42 ± 0.07	0.54 ± 0.13	0.59 ± 0.10	3.50 ± 0.94	4.07 ± 0.52
Σ SFA	40.4 ± 0.58	40.5 ± 0.54	36.6 ± 0.73	37.5 ± 0.77	32.3 ± 2.85	30.4 ± 2.30	38.4 ± 1.50	39.4 ± 1.86	32.7 ± 3.05	32,5 ± 1.51	34.6 ± 1.02	35.4 ± 1.30	33.9 ± 1.03	34.1 ± 1.08
16:1	1.18 ± 0.34	1.29 ± 0.22	2.77 ± 1.21	2.70 ± 0.62	1.97 ± 1.12	1.56 ± 0.33	8.81 ± 1.10	9.27 ± 0.97	7.83 ± 2.58	7.44 ± 1.67	10.0 ± 2.01	9.55 ± 1.50	0.43 ± 0.11	0.48 ± 0.04
18:1n-9	5.74 ± 0.50	6.05 ± 0.51	6.19 ± 0.96	5.70 ± 0.50	5.99 ± 1.64	6.09 ± 1.01	22.9 ± 2.12	21.7 ± 1.16	22.7 ± 1.87	21.3 ± 1.33	23.4 ± 1.77	21.9 ± 1.50	26.5 ± 0.57	26.9 ± 1.05
18:1n-7	3.29 ± 0.28	3.50 ± 0.49	4.53 ± 0.66	4.18 ± 0.93	4.67 ± 0.54	4.75 ± 1.09	3.62 ± 0.29	3.42 ± 0.26	4.58 ± 0.38	4.38 ± 0.47	4.33 ± 0.38	4.12 ± 0.23	nd	nd
Other MUFA	0.76 ± 0.16	0.71 ± 0.07	0.39 ± 0.20	0.38 ± 0.04	0.33 ± 0.13	0.31 ± 0.05	0.44 ± 0.13	0.43 ± 0.09	0.97 ± 0.37	0.96 ± 0.17	0.80 ± 0.12	0.87 ± 0.15	9.77 ± 0.74	11.2 ± 1.96
Σ MUFA	11.6 ± 0.93	12.0 ± 0.78	13.9 ± 2.67	13.0 ± 1.86	12.6 ± 2.60	12.8 ± 2.00	35.8 ± 2.85	34.8 ± 1.66	36.1 ± 3.87	34.1 ± 2.46	38.6 ± 3.90	36.4 ± 2.62	37.7 ± 2.26	38.5 ± 2.51
18:2n-6 (LA)	10.1 ± 0.46	10.5 ± 0.68	16.0 ± 1.35	16.1 ± 0.93	24.8 ± 2.71	26.4 ± 3.40	14.8 ± 2.54	15.2 ± 1.88	26.7 ± 4.01	27.5 ± 1.61	21.9 ± 3.36	22.3 ± 1.68	nd	nd
18:3n-3 (ALA)	0.06 ± 0.03	0.09 ± 0.01	0.16 ± 0.13	0.16 ± 0.02	nd	nd	0.60 ± 0.09	0.70 ± 0.13	1.33 ± 0.09	1.49 ± 0.06	1.25 ± 0.11	1.34 ± 0.13	nd	nd
20:4n-6 (AA)	23.0 ± 1.03	20.5 ± 0.80 *	20.5 ± 1.86	18.7 ± 0.71 *	14.2 ± 2.38	14.0 ± 1.23	6.95 ± 1.86	6.23 ± 0.76	0.69 ± 0.30	0.66 ± 0.27	0.59 ± 0.75	0.35 ± 0.06	7.13 ± 0.30	6.88 ± 0.71
20:5n-3 (EPA)	0.59 ± 0.07	1.32 ± 0.11 *	0.93 ± 0.14	2.11 ± 0.19 *	0.19 ± 0.04	0.50 ± 0.12 *	0.12 ± 0.07	0.19 ± 0.16	0.06 ± 0.05	0.21 ± 0.04 *	0.04 ± 0.03	0.22 ± 0.07 *	nd	nd
22:5n-3 (DPA)	1.65 ± 0.16	2.32 ± 0.10 *	1.17 ± 0.28	2.05 ± 0.25 *	1.26 ± 0.29	1.48 ± 0.58	0.26 ± 0.08	0.26 ± 0.08	0.12 ± 0.11	0.18 ± 0.14	0.05 ± 0.08	0.14 ± 0.10	nd	nd
22:6n-3 (DHA)	3.84 ± 0.25	3.73 ± 0.25	6.65 ± 0.89	6.44 ± 0.42	7.47 ± 1.83	7.94 ± 0.97	0.68 ± 0.20	0.70 ± 0.11	0.39 ± 0.14	0.47 ± 0.06	0.22 ± 0.14	0.40 ± 0.27	10.9 ± 0.99	10.4 ± 1.00
Other PUFA	1.66 ± 0.27	1.63 ± 0.09	1.32 ± 0.42	1.62 ± 0.11	1.46 ± 0.34	1.85 ± 0.77	1.45 ± 0.06	1.56 ± 0.27	1.92 ± 0.67	1.72 ± 0.46	1.72 ± 0.93	1.63 ± 0.83	8.67 ± 0.37	8.87 ± 0.65
Σ PUFA	43.2 ± 1.37	42.6 ± 1.05	46.6 ± 2.70	47.0 ± 1.27	49.3 ± 5.92	52.4 ± 2.04	24.6 ± 2.07	24.7 ± 1.94	30.1 ± 4.62	31.4 ± 1.72	25.4 ± 4.20	25.9 ± 1.79	26.7 ± 1.12	26.2 ± 1.39
Σ n-3	9.05 ± 0.28	10.4 ± 0.25	9.39 ± 1.11	11.2 ± 0.51 *	9.10 ± 1.80	11.5 ± 1.19 *	1.82 ± 0.26	2.02 ± 0.35	1.97 ± 0.47	2.48 ± 0.21	1.87 ± 0.50	2.05 ± 0.41	17.9 ± 0.81	17.7 ± 0.72
Σ n-6	33.7 ± 1.31	31.6 ± 0.99	37.0 ± 2.93	35.5 ± 1.42	39,2 ± 4.52	40.2 ± 2.87	21.8 ± 2.77	21.5 ± 1.90	27.7 ± 4.34	28.5 ± 1.71	23.0 ± 3.88	23.4 ± 1.74	8.53 ± 0.48	8.22 ± 0.76
Σ n-3/Σn-6	0.27 ± 0.01	0.33 ± 0.01 *	0.26 ± 0.04	0.32 ± 0.02 *	0.23 ± 0.04	0.29 ± 0.05	0.08 ± 0.01	0.09 ± 0.01	0.07 ± 0.01	0.09 ± 0.01 *	0.08 ± 0.02	0.09 ± 0.02	2.10 ± 0.10	2.16 ± 0.14

Other SFA: 15:0, 15:0iso, 16:0iso, 17:0, 17:0iso, 19:0, 20:0, 22:0, 24:0; Other MUFA: 17:1, 20:1n-9, 20:1n-7, 22:1n-11, 22:1n-9, 24:1n-9; Other PUFA: 16:2n-4, 16:3n-4, 16:3n-3, 16:4n-3, 18:3n-6, 18:4n-3, 20:2n-6, 20:4n-3; 22:5n-6; nd–not detected; * *p* < 0.05.
